# Differential genes expression analysis of invasive aspergillosis: a bioinformatics study based on mRNA/microRNA

**DOI:** 10.22099/mbrc.2020.37432.1509

**Published:** 2020-12

**Authors:** Maryam Hosseinipour, Shirin Shahbazi, Shahla Roudbar-Mohammadi, Maryam Khorasani, Majid Marjani

**Affiliations:** 1 Department of Medical Mycology, Faculty of Medical Science, Tarbiat Modares University, Tehran Iran; 2 Department of Medical Genetics, Faculty of Medical Sciences, Tarbiat Modares University, ‎Tehran, Iran; 3 Molecular Medicine Department, Pasteur Institute of Iran, Tehran, Iran; 4 Clinical Tuberculosis and Epidemiology Research Center, National Research Institute of Tuberculosis and Lung Diseases, Shahid Beheshti University of Medical Sciences, Tehran, Iran

**Keywords:** Fungal infection, Gene expression, MicroRNAs, Signaling pathways

## Abstract

Invasive *aspergillosis* is a severe opportunistic infection with high mortality in immunocompromised patients. Recently, the roles of microRNAs have been taken into consideration in the immune system and inflammatory responses. Using bioinformatics approaches, we aimed to study the microRNAs related to invasive *aspergillosis *to understand the molecular pathways involved in the disease pathogenesis. Data were extracted from the gene expression omnibus (GEO) database. We proposed 3 differentially expressed genes; S100B, *TDRD9* and *TMTC1* related to pathogenesis of invasive *aspergillosis*. Using miRWalk 2.0 predictive tool, microRNAs that targeted the selected genes were identified. The roles of microRNAs were investigated by microRNA target prediction and molecular pathways analysis. The significance of combined expression changes in selected genes was analyzed by ROC curves study. Thirty-three microRNAs were identified as the common regulator of *S100B*, *TDRD9* and *TMTC1* genes. Several of them were previously reported in the pathogenesis of fungal infections including miR-132. Predicted microRNAs were involved in innate immune response as well as toll-like receptor signaling. Most of the microRNAs were also linked to platelet activation. The ROC chart in the combination mode of *S100B/TMTC1*, showed the sensitivity of 95.65 percent and the specificity of 69.23 percent. New approaches are needed for rapid and accurate detection of invasive *aspergillosis*. Given the pivotal signaling pathways involved, predicted microRNAs can be considered as the potential candidates of the disease diagnosis. Further investigation of the microRNAs expression changes and related pathways would lead to identifying the effective biomarkers for IA detection.

## INTRODUCTION

Invasive *aspergillosis* (IA) exhibits more than 80 percent mortality rate in individuals with immunodeficiency, including patients with blood malignancies and bone marrow transplant recipients. The incidence of IA has not been well elucidated yet, however it was considered responsible for 30-50 percent of invasive fungal diseases among immunocompromised patients [1] . *Aspergillus fumigatus* and *Aspergillus flavus* are the most common cause of IA [2]. The diagnosis is mainly based on clinical examinations and serological tests. The gold standard methods are histopathological tests and tissue culture following the lung biopsy or bronchoalveolar lavage (BAL). However, this invasive approach is contraindicated in severe conditions such as thrombocytopenia [3]. Since IA progresses rapidly, the high mortality rate is a great challenge due to the lack of prompt standard diagnostic test. 

Recently, the role of microRNAs has been taken into consideration as small molecules that are involved in the immune system and inflammatory response [4]. MicroRNAs regulate the gene expression following the external stimuli. Expression and function of microRNAs are essential for numerous physiological functions and cellular homeostasis. Changes in microRNAs can affect the expression of several target genes and subsequent proteins [5]. 

Evaluation of the mRNAs/microRNAs levels would lead to the identification of the key factors in pathways that are involved in the disease pathogenesis [6]. Active cells produce microRNAs that can be detected and traced in body fluids. As a result, circulating microRNAs are potential biomarkers in a variety of diseases, such as cancer, metabolic disorders, and cardiovascular diseases [7]. 

Following infection, significant changes occur in the profiles of circulating microRNAs [8]. It has been shown that the expression of miR-455, miR-125a, miR-146 and miR-155 were increased in rat macrophages in response to *Candida albicans* infection [9]. The expression of miR-204 and miR-211 were decreased in kidney tissue of rats with candidemia-induced kidney injuries [10]. In monocytes and dendritic cells contaminated with *Aspergillus fumigatus*, miR-132 and miR-155 showed higher expression levels [11]. Serum analysis of the patients infected with *P. brasiliensis* revealed increased expression of 8 microRNAs linked to apoptosis and immune response [12]. 

Validation of clinical biomarkers is a pivotal aspect in bioinformatics and biostatistics. With the development of high-power technologies, profiling the multiple gene expression is a useful approach to find differentially expressed genes correlated to the disease pathogenesis. Since microRNAs are quite stable in different ranges of clinical specimens, they could serve as biomarkers [13]. 

Based on this knowledge, we aimed to investigate the microRNAs that could be applied as the disease biomarkers. Given the vitality of early diagnosis of IA, we analyzed the available datasets using various bioinformatics tools to find the microRNAs most connected to the pathogenesis of the disease.

## MATERIALS AND METHODS

**Microarray and published data used for gene selection: **In the present study, the gene expression dataset GSE78000 with the platform of Affymetrix Human Genome 19 (GPL21464) was extracted from the gene expression omnibus (GEO) database (https://www.ncbi.nlm. nih.gov/gds). GSE78000 included 23 samples obtained from haematological patients with IA and 13 samples from non-IA haematological patients. Two of the non-IA samples were reported as a possible invasive fungal disease (IFD). Nine control samples from healthy donors were also included in the dataset. The S100 calcium-binding protein B *(S100B)* was suggested as a potential new biomarker for the diagnosis of IA on the GSE78000 [14]. Recently, using the same dataset, transmembrane O-mannosyltransferase targeting cadherins* 1TMTC1* gene was introduced as a new biomarker of IA [15]. Since IA shares many in commons with severe inﬂammatory response syndrome we also included tudor domain containing 9 *(TDRD9)* gene in our study. Using microarray analysis *TDRD9* was previously identiﬁed related to the pathogenesis of the SIRS [16]. 

**Identification of gene targeting microRNAs: **The predicted microRNAs that target *S100B*,* TDRD9* and *TMTC1* were identified using the predictive tool, miRWalk 2.0 (http://zmf. umm.uni-heidelberg.de/apps/zmf/mirwalk2/) [17]. To confirm the obtained results additional bioinformatics algorithms were applied including, miRNAMap, RNA22, MicroT4, miRanda, RNAhybrid, PICTAR2, miRBridge, miRWalk, PITA, miRDB, miRMap, and Targetscan. 

**In-silco pathway analysis: **The roles of microRNAs in molecular pathways were evaluated based on the Kyoto encyclopedia of genes and genomes (KEGG). Analysis of gene ontology (GO) was examined using the DIANA TOOLS-mirPath v.3 database (http://snf-515788.vm. okeanos.grnet.gr/).

**Analysis of the ROC curve: **MedCalc V.12.1.4 software was applied to analyze the significance of expression change in selected gene by drawing the ROC curves. The gene expression data were extracted from GSE78000 dataset. A logistic regression model was used to check the combination modes of gene expressions. The area under the curve, sensitivity and one minus its specificity were calculated to compare the predictive values of the genes. 

## RESULTS

According to the analysis of microRNAs, predicted by the miRWalk, 33 microRNAs were able to target *S100B*, *TDRD9* and *TMTC1* (Table 1). To this end, microRNAs approved by at least three different algorithms were considered significant. The sequences of the microRNAs have been indicated in Table 1. One of the predicted microRNAs, miR-132, was previously shown related to *Aspergillus *infection. Our list also comprised microRNAs with a known function in fungal infection such as miR-155. However, we also found microRNAs that had not been previously reported to be associated with infection or inflammation. 

**Table 1:** The microRNAs predicted by miRWalk2.0 with ability to target *S100B*, *TMTC1* and *TDRD9*

**ID**	**Accession **	**Sequence**
hsa-miR-516a-3p	MIMAT0006778	UGCUUCCUUUCAGAGGGU
hsa-miR-516b-3p	MIMAT0002860	UGCUUCCUUUCAGAGGGU
hsa-miR-1287-5p	MIMAT0005878	UGCUGGAUCAGUGGUUCGAGUC
hsa-miR-583	MIMAT0003248	CAAAGAGGAAGGUCCCAUUAC
hsa-miR-3978	MIMAT0019363	GUGGAAAGCAUGCAUCCAGGGUGU
hsa-miR-186-5p	MIMAT0000456	CAAAGAAUUCUCCUUUUGGGCU
hsa-miR-490-5p	MIMAT0004764	CCAUGGAUCUCCAGGUGGGU
hsa-miR-155-5p	MIMAT0000646	UUAAUGCUAAUCGUGAUAGGGGUU
hsa-miR-4717-5p	MIMAT0019829	UAGGCCACAGCCACCCAUGUGU
hsa-miR-650	MIMAT0003320	AGGAGGCAGCGCUCUCAGGAC
hsa-miR-345-5p	MIMAT0000772	GCUGACUCCUAGUCCAGGGCUC
hsa-miR-551b-5p	MIMAT0004794	GAAAUCAAGCGUGGGUGAGACC
hsa-miR-875-3p	MIMAT0004923	CCUGGAAACACUGAGGUUGUG
hsa-miR-576-5p	MIMAT0003241	AUUCUAAUUUCUCCACGUCUUU
hsa-miR-593-3p	MIMAT0004802	UGUCUCUGCUGGGGUUUCU
hsa-miR-3928-3p	MIMAT0018205	GGAGGAACCUUGGAGCUUCGGC
hsa-miR-346	MIMAT0000773	UGUCUGCCCGCAUGCCUGCCUCU
hsa-miR-7856-5p	MIMAT0030431	UUUUAAGGACACUGAGGGAUC
hsa-miR-7162-5p	MIMAT0028234	UGCUUCCUUUCUCAGCUG
hsa-miR-222-3p	MIMAT0000279	AGCUACAUCUGGCUACUGGGU
hsa-miR-1276	MIMAT0005930	UAAAGAGCCCUGUGGAGACA
hsa-miR-383-5p	MIMAT0000738	AGAUCAGAAGGUGAUUGUGGCU
hsa-miR-1289	MIMAT0005879	UGGAGUCCAGGAAUCUGCAUUUU
hsa-miR-4311	MIMAT0016863	GAAAGAGAGCUGAGUGUG
hsa-miR-34c-3p	MIMAT0004677	AAUCACUAACCACACGGCCAGG
hsa-miR-4652-3p	MIMAT0019717	GUUCUGUUAACCCAUCCCCUCA
hsa-miR-384	MIMAT0001075	AUUCCUAGAAAUUGUUCAUA
hsa-miR-4743-3p	MIMAT0022978	UUUCUGUCUUUUCUGGUCCAG
hsa-miR-887-3p	MIMAT0004951	GUGAACGGGCGCCAUCCCGAGG
hsa-miR-132-3p	MIMAT0000426	UAACAGUCUACAGCCAUGGUCG
hsa-miR-642a-5p	MIMAT0003312	GUCCCUCUCCAAAUGUGUCUUG
hsa-miR-2115-5p	MIMAT0011158	AGCUUCCAUGACUCCUGAUGGA
hsa-miR-34b-3p	MIMAT0004676	CAAUCACUAACUCCACUGCCAU

We investigated statistical significant roles of microRNAs in KEGG pathways which is a reference database for pathway mapping. The results revealed regulatory roles of the microRNAs in several signaling pathways with the highest significance related to mucin type O-Glycan biosynthesis (P<0.05) (Table 2). Several other important pathways also explored including proteoglycans in cancer. It should be noted that many pathogens recruit proteoglycans to invade host cells.

**Table 2:** Results of examining KEGG of microRNAs predicted by mirParth v.3

**KEGG pathway**	**P-Value**	**#genes**	**#miRNAs**
Mucin type O-Glycan biosynthesis	1.22E-06	15	14
Proteoglycans in cancer	2.50E-06	110	28
GABAergic synapse	2.76E-06	45	26
Signaling pathways regulating pluripotency of stem cells	3.15E-06	79	28
Hippo signaling pathway	1.18E-05	83	29
Renal cell carcinoma	1.86E-05	43	28
Prion diseases	3.44E-05	12	12
Glioma	0.0001508	38	28
Pathways in cancer	0.0001508	200	32
Circadian rhythm	0.0001865	23	24
Wnt signaling pathway	0.0003638	73	28
FoxO signaling pathway	0.0005044	74	26
Adrenergic signaling in cardiomyocytes	0.0009892	76	31
Long-term potentiation	0.001245	42	28
AMPK signaling pathway	0.0019863	69	30
Glutamatergic synapse	0.0023066	59	30
cAMP signaling pathway	0.0023066	104	31
Gap junction	0.0025114	45	29
Nicotine addiction	0.0026905	25	22
Prostate cancer	0.0026905	50	31
Estrogen signaling pathway	0.0028283	50	29
cGMP-PKG signaling pathway	0.0028422	85	31
Rap1 signaling pathway	0.0029022	105	30
Thyroid hormone synthesis	0.0037497	36	28
Axon guidance	0.0039922	63	25
Alanine, aspartate and glutamate metabolism	0.0042956	22	21
Long-term depression	0.0046957	32	28
ErbB signaling pathway	0.0046957	49	29
MAPK signaling pathway	0.0046957	126	30
PI3K-Akt signaling pathway	0.0046957	161	32
Gastric acid secretion	0.004872	43	25
Ubiquitin mediated proteolysis	0.0052406	74	29
Insulin secretion	0.0052406	47	29
Oocyte meiosis	0.0055651	62	30
Oxytocin signaling pathway	0.0057047	80	29
Amphetamine addiction	0.0063306	36	29
SNARE interactions in vesicular transport	0.0089967	20	24
Protein processing in endoplasmic reticulum	0.0112118	81	29

**Figure 1:**
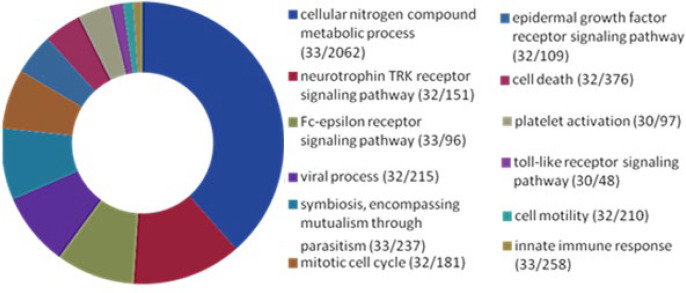
Pie chart the biological processes analysis of predicted microRNAs. The first number in parentheses indicates the number of microRNAs, and the second number in parentheses indicates the number of genes involved

As indicated in Figure 1, the results of the study on GO of microRNAs using mirPath v.3 revealed that the all 33 predicted microRNAs were involved in the innate immune response. Thirty of them were linked to the toll-like receptor (TLR) signaling. Furthermore, most of the microRNAs play role in platelet activation. These fundamental functions contribute in the pathophysiologic process of IA. 

**Figure 2:**
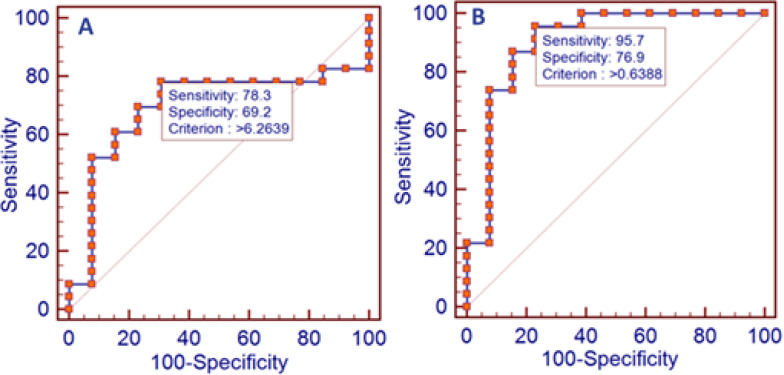
ROC curve analysis to evaluate the diagnostic value of *S100B*, *TMTC1* and *TDRD9* expression in the IA. **A:** Analysis of the ROC curve for *S100B*/*TMTC1*/*TDRD9* combination. **B:** Analysis of the ROC curve for *S100B*/*TMTC1* gene expression data combination

The ROC curve analyses were shown in Figure 2. The combined panel of three genes, *S100B/ TDRD/ TMTC1* could detect the IA with AUC: 0.69, sensitivity: 78.26, and specificity: 69.23. Statistical analysis showed that 95 percent confidence interval (CI) of combined 3 genes was 0.520 to 0.837 with the significance P value of 0.04. Meanwhile, the ROC chart had robust results in the combination mode of *S100B/TMTC1* with AUC: 0.9, sensitivity: 95.65, and specificity: 69.23. The reported CI was 0.757 to 0.976 and P value was calculated <0.0001 (Fig. 2).

## DISCUSSION

*Aspergillus fumigatus* and *Aspergillus flavus* are saprophyte fungi, widespread in the environment. Exposure to fungal spores leads to IA in immunocompromised patients, with a high mortality rate [18]. Studies to find new biomarkers for rapid and accurate detection of IA are ongoing. Recently, triacetylfusarinine C which is an *Aspergillus* fumigatus siderophore was introduced as a urine biomarker for early diagnosis of IA [19]. Furthermore, high-throughput screening and bioinformatics studies have been conducted to identify diagnostic biomarkers in various diseases including IA [20]. Comparing gene expression profiles of IA with non-IA patients, it has been shown that *S100B* could be served as a diagnostic biomarker of IA [14].

*TMTC1* was up regulated 2.6 folds in IA comparing to non-IA patients with the 78.3 percent sensitivity and 81.8 percent specificity [15]. *TMTC1* is located on the membrane of endoplasmic reticulum and play a role in calcium homeostasis. It is also involved in the protein glycosylation by mannosyl transfer to the hydroxyl group of serine or threonine residues [21].

On the other hand, *TDRD9* is a DEXH-box RNA helicase and is involved in PIWI-interacting RNAs (piRNAs) formation [22]. Besides the male reproductive system, it mainly expressed in blood cells including monocytes and dendritic cells which play important roles in the innate immune response against IA. Monocytes express a variety of receptors for the identification of fungal cells, such as TLRs, c-type lectin receptors (CLRs) and dectin-1. These receptors detect fungal pathogen molecules such as beta-d-glucan that are located in the cell wall of *Aspergillus* species.

In our study, we identified 33 microRNAs as the regulator of *S100B*, *TDRD9* and *TMTC1*. Based on our finding, predicted microRNAs were involved in key cellular functions including TLR signaling [23]. It has been shown that innate immune detection of *Aspergillus fumigatus* is facilitated by TLRs [24]. 

Our results also revealed that all 33 microRNAs were involved in innate immune response. Among them, miR-132 was previously recognized related to the *Aspergillus *infection. Gupta *et al.* showed that miR-132 is differentially expressed in monocytes and dendritic cells following contamination by *Aspergillus fumigatus* [11]. As mentioned earlier, monocyte and dendritic cells are among the main expression sites of *TDRD9* which is a conserved target of miR-132. New roles for *TDRD9* have also been identified in lung cancer and was suggested as a potential therapeutic target [25]. Furthermore, miR-132 was reported to be increased in dendritic cells and natural killer cells following the exposure to *Aspergillus fumigatus* [26]. 

The microRNAs predicted in our study also included miR-155 which is a negative regulator of TLRs [27]. It has been shown that miR‐155 is an essential factor in the innate immune response to fungal infection [28]. 

We also observed that the predicted microRNAs were related to platelet activation process. The activation of Platelets is an important component of hemostasis and *Aspergillus fumigatus* is a well-known platelets activator [29]. On the other hand, it has been elucidated that platelets are important factors in tissue integrity following pulmonary infection of *Aspergillus fumigatus* [30]. 

Previous studies have indicated that microRNAs regulate the host response in viral, fungal, and bacterial infections [31]. Although the pathogenesis of IA is not well known, many factors such as microRNAs may contribute to the disease development. Also, understanding molecular pathways involved in the disease pathogenesis could lead to the finding of new biomarkers [32]. According to the result of the present study, further evaluation of 33 predicted microRNAs can lead to the design of a diagnostic panel for IA. Analyses of differentially expressed microRNAs are a promising approach to improve the proper diagnosis of the condition and could lead to a better understanding of the mechanisms underlying the association between human host cells and IA. 
